# Topological decomposition and transformation of photonic quasicrystals

**DOI:** 10.1515/nanoph-2025-0384

**Published:** 2025-10-30

**Authors:** Hao Wang, Houan Teng, Jinzhan Zhong, Xinrui Lei, Qiwen Zhan

**Affiliations:** School of Optical-Electrical and Computer Engineering, 47863 University of Shanghai for Science and Technology, Shanghai, 200093, China; Zhejiang Key Laboratory of 3D Micro/Nano Fabrication and Characterization, Department of Electronic and Information Engineering, School of Engineering, Westlake University, Hangzhou, Zhejiang, 310030, China; Zhangjiang Laboratory, 100 Haike Road, Shanghai, 201204, China

**Keywords:** photonic skyrmion, spin quasicrystal, surface plasmon polariton

## Abstract

Photonic quasicrystals, generated through the interference of multiple vortex beams, exhibit rich and complex topological landscapes. However, unlike their periodic counterparts, they have far lacked the same level of controllability and reconfigurability. In this work, we develop a theoretical model to characterize the spin topology of photonic quasicrystals and uncover the intrinsic substructure underlying their quasi-periodic spin textures. By analyzing the formation mechanisms, we demonstrate the controlled decomposition and topological annihilation of individual sublattices within a quasicrystalline configuration. Based on this, we propose a phase-modulation method to reconfigure these topological states. We demonstrate that a quasicrystal with octagonal symmetry can be decomposed into two square meron lattices with a relative twist. This method is further extended to create more complex quasicrystals, where selective sublattice activation leads to meron bags. These findings provide new insights into both the static design and active manipulation of topological quasicrystals of light, paving the way for programmable topological photonic platforms with high spatial complexity and functional versatility.

## 1 Introduction

Topological quasiparticles are localized field configurations that exhibit stable, particle-like properties. The exploration of topological quasiparticles has emerged as a central topic in modern physics, bridging condensed matter physics, high-energy physics, and photonics [1], [2], [3], [4], [5], [6], [7], [8], [9], [10]. These quasiparticles, such as magnetic skyrmion [11], [12], [13], [14], and vortices in superfluids [15] exhibit remarkable stability against local perturbations, making them ideal candidates for robust information carriers and novel computing paradigms [16], [17], [18], [19], [20]. In recent years, such topological quasiparticles have been observed in the realm of optics. By precisely sculpturing the phase, polarization, and amplitude of light, optical analogues of magnetic skyrmions are constructed by either spin angular momentums (SAMs) [21], [22], [23], [24], [25], [26], [27], electromagnetic field [28], [29], [30], [31], [32], [33], Stokes vectors [34], [35], [36], [37], [38], [39] or Poynting vectors [40], [41], offering flexible control and direct observability under ambient conditions. This allows for advanced applications in optical communications, quantum information processing [42], [43], [44], and ultra-precise metrology [5], [45], [46], [47], [48].

Similar to magnetic skyrmions in condensed matter systems, photonic skyrmions can exist either as isolated entities or periodic arrays (skyrmion lattices), depending on the spatial distribution of the optical field, the symmetry and geometry of the excitation pattern, and the boundary conditions of the medium. Isolated photonic skyrmions typically arise from localized phase or polarization singularities, while periodic skyrmion lattices emerge from interference patterns or periodic modulation of structured light – that enforce topological order across space. Typically, the interfering patterns can only be regularly tessellated with 3, 4 or 6-folds rotational symmetries in two dimensions, giving rise to nontrivial skyrmion lattices, where the skyrmion or meron topology is constrained by the symmetry of optical field [23], [49], [50], [51], [52]. However, when the symmetry of the interfering system deviates from these conventional crystallographic symmetries, for instance, for *N* = 5, 7 or other non-crystallographic values, the system enters the fascinating realm of quasicrystals [53], [54]. These topological quasicrystals exhibit long-range order but lack the translational periodicity of their crystalline counterparts, allowing for rotational symmetries forbidden in conventional crystals and potentially much higher topological complexity.

The study of topological quasicrystals is a rapidly emerging frontier. Significant progress has been made in exploring their generation and the characterization of their distinct Fourier spectra, revealing intricate, layered substructures [53], [54], [55], imparting them with novel propagation dynamics [56]. While these efforts confirm the existence of such exotic topological states, two significant challenges remain. First, a systematic theoretical framework capable of analytically predicting the topology and internal structure of photonic quasicrystals is still in its infancy. Second, and more critically, these complex topological structures have so far been static. The development of methods to fundamentally reconfigure the internal topology of a quasicrystal – for instance, by transforming its constituent sublattices into a periodic crystallographic lattice or by synthesizing novel, artificial textures on demand – has not yet been demonstrated. Overcoming these challenges is essential for unlocking the potential of topological quasicrystals in functional photonic devices.

**Figure 1:**
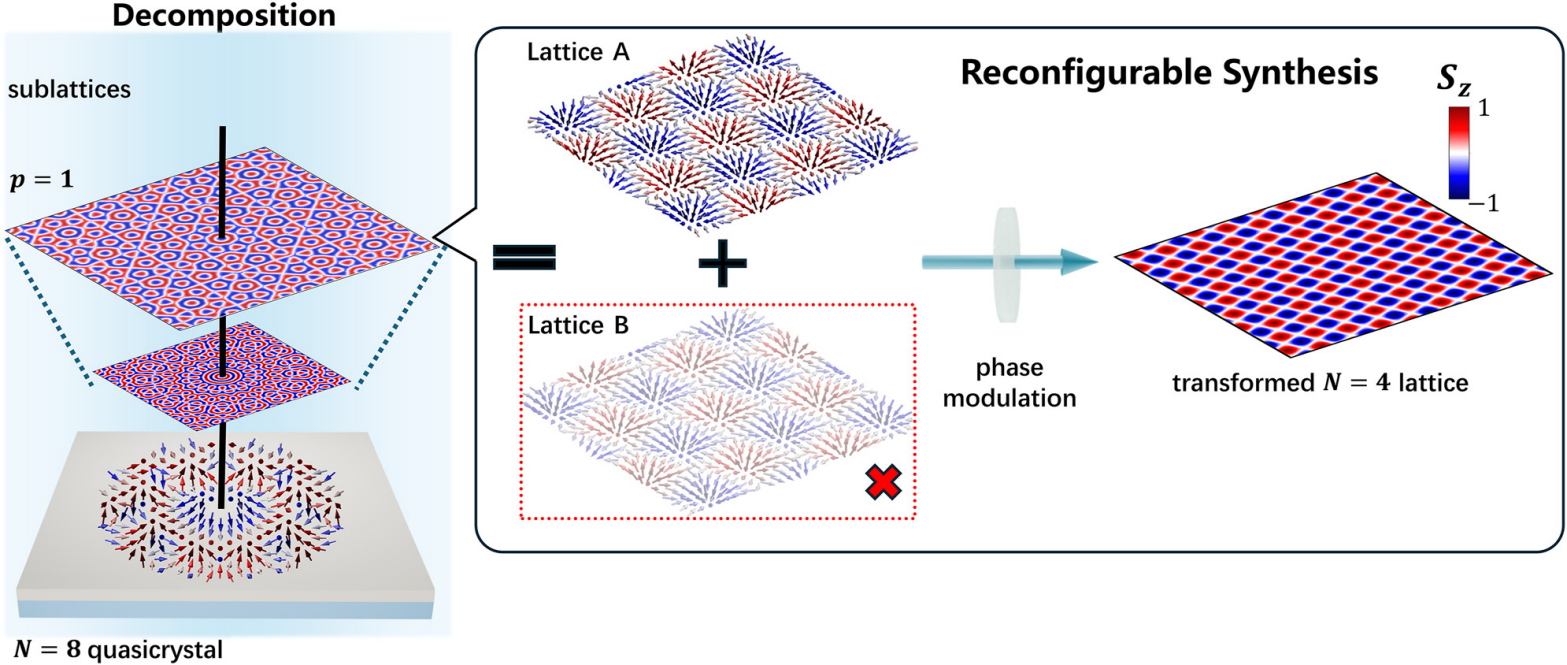
Conceptual schematic of the decomposition and reconfigurable synthesis of a topological quasicrystal of light. The process begins with an *N* = 8 spin quasicrystal. Through analytical decomposition, the quasicrystal with octagonal symmetry is separated into its constituent periodic sublattices, which can be considered as a superposition of two fundamental meron lattices distinguished by a relative angular twist (Lattice A and B). By applying a tailored phase mask, we can selectively annihilate one of these components (Lattice B), and the spin quasicrystal is transformed into a periodic topological spin lattice.

In this work, we propose a theoretical framework for the generation, reconfigurable decomposition and synthesis of topological quasicrystals. By analyzing the formation mechanism and intrinsic substructure of the quasi-periodic photonic spin textures, we demonstrate the controlled composition and topological annihilation of individual sublattices within the quasicrystalline configuration. Building on this foundation, we construct a novel phase-modulation scheme to achieve reconfigurable control over these topological states. As a representative example, we show that a spin quasicrystal with octagonal symmetry can be decomposed into two meron lattices with square symmetry, distinguished by a relative angular twist (Figure 1). This principle is further generalized to engineer higher order quasicrystals, where selective activation of specific constituent sublattices leads to the formation of meron bags-topologically rich structures that allow for on-demand manipulation of topological charge density [57], [58], [59]. While we focus on SPP-based systems as a prime example, the fundamental principles and design rules developed here are broadly applicable to free space and other wave platforms. These findings provide novel insight for the static design and active manipulation of topological quasicrystals of light, paving the way for programmable topological photonic systems with high spatial complexity and functional versatility.

## 2 Theoretical framework

### 2.1 Formation and decomposition mechanism of spin quasicrystals

**Figure 2:**
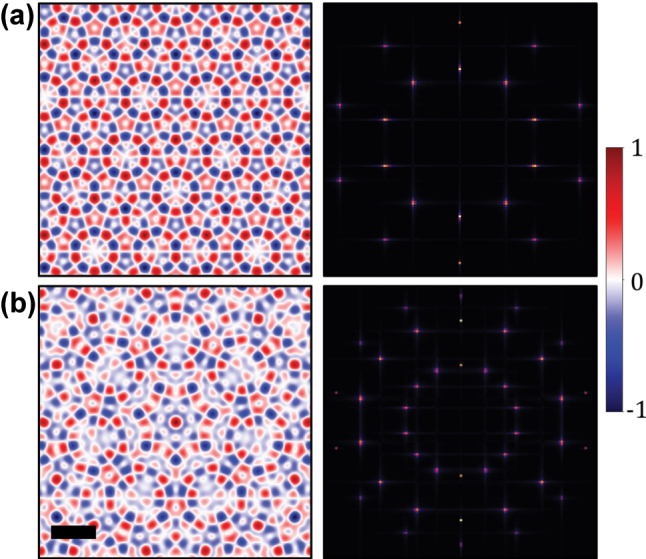
Representative examples of spin quasicrystals. Spin textures for (a) *N* = 5 and (b) *N* = 7 quasicrystals, both generated with the interaction between an OAM beam (*L* = 1) and polygonal metallic nanoslit. The texture patterns depict complex, non-periodic structure and long-range-order, compared with skyrmionic lattice. The corresponding Fourier domain spectra are depicted on right panels, comprised of discrete concentric rings revealing the sublattice structure. The scale bar shown on (b) corresponds to SPP wavelength *λ*_*r*_.

The formation of spin quasicrystals can be modeled by considering the coherent interference of multiple surface plasmon polariton (SPP) plane waves. Experimentally, these waves can be excited by illuminating a coupling structure, typically a polygonal slit milled in a thin metal film. For the polygonal slit with *N*-fold rotational symmetry, each slit segment functions as a nanoantenna that couples free space light into surface wave. Due to the symmetry constraints imposed by the structure, *N* SPP waves are excited and propagating in equally separated direction $\theta_{m}=\frac{2\pi m}{N}$ (where *m* = 1, 2,…*N*). The electromagnetic field arising from the spin–orbit interaction of light can be described by a scalar Hertz potential Ψ as [60]:

(1) $$\Psi =\overset{N}{\underset{m=1}{\sum }}A_{0}e^{iL\theta_{m}}{\text{e}}^{\text{i}k_{r}\left(x\cos\theta_{m}+y\sin\theta_{m}\right)}e^{-k_{z}z}$$

where *A*_0_ is a constant, *L* is the OAM of the incident beam, *k*_*r*_ and *k*_*z*_ are the transverse and longitudinal wave vector component satisfying $k_{0}^{2}=k_{r}^{2}-k_{z}^{2}$ with *k*_0_ being the free-space wavenumber. The term $e^{iL\theta_{m}}$ represents the helical phase inherited from the incident OAM beam. The electromagnetic fields **E** and **H** can be obtained accordingly [23], [61]. The local spin density **S** is calculated through the vector products of the physical fields, which can be expressed in terms of Berry curvature of Hertz potential as [62], [63], [64]:

(2) $$S=\frac{\varepsilon k_{r}^{2}}{4\omega }\text{Im}\left(\nabla \Psi^{*}\times \nabla \Psi \right)$$

where ∗ indicates complex conjugation, *ω* denotes the angular frequency of the electromagnetic field; *ɛ* is the permittivity of the medium, respectively. Skyrmion and meron lattices are generated from Equations (1) and (2) by imposing translational and rotational symmetry of the Hertz potential for *N* = 3,4,6. While for other values of *N*, the Hertz potential lacks translational symmetry, leading to the formation of spin quasicrystals. The longitudinal components of SAM in photonic spin quasicrystals with *N* = 5 and *N* = 7 are illustrated in Figure 2, where the spin textures reveal intricate long-range order without periodic repetition. These patterns can be fundamentally interpreted as linear superpositions of multiple sublattices, each characterized by distinct lattice constants and orientations.

To reveal the sublattice configurations, the longitudinal component of the spin density is calculated from Equations (1) and (2) as

(3) $$S_{z}\propto \underset{m}{\sum }\underset{n}{\sum }\sin\left(Tp\right)\cdot \sin\left[K_{m-n}\left(m,n\right)\cdot r+\phi_{p}\right]$$

where the indices *m* and *n* (ranging from 1 to *N*) label the individual interfering SPP waves excited from the *N* segments of the polygonal slit, *T* = 2*π*/*N*, $K_{m-n}=k_{r}\left(\cos\theta_{m}-\cos\theta_{n},\sin\theta_{m}-\sin\theta_{n}\right)$ , *ϕ*_*p*_ = *LTp*.The entire spin texture is thus a result of the pairwise interference between all possible wave combinations (*m*, *n*). To distinguish different combinations, we introduce a relative index *p* = *m*−*n*, which serves as the order or index of each interference sublattice. Equation (3) represents a summation over a series of fundamental interference patterns, each defined by a unique wavevector $\left|K_{p}\left(m,n\right)\right|=2\left|\sin\frac{\pi }{N}p\right|k_{r}$ , and corresponding phase *ϕ*_*p*_. The wavevector **K**_*p*_ represents the position in the Fourier spectrum, confirming that the complexity of the quasicrystal is governed by the discrete set of allowed interference wavevectors, determined by *N* and *p*. Among different sublattice layers, their lattice constants can be calculated from the magnitude of wavevector.

The decomposition of topological spin lattice can be illustrated in Fourier space. The Fourier spectra of the photonic spin quasicrystals with *N* = 5 and *N* = 7 are depicted in the inset of Figure 2, which consist of several discrete, concentric rings, confirming the sublattice structure. In the Fourier space, each ring, comprising *N* distinct points, corresponds to a set of wavevectors **K**_*p*_ that share identical magnitude as predicted by Equation (3). The global spin texture is the result of interplay between different sublattices, with each sublattice *C*_*p*_ formed by the coherent superposition of all interference terms that share the same wavevector magnitude **|K**_*p*_|. By grouping common terms with the degeneracy (**|K**_*p*_| = **|K**_−*p*_| = **|K**_*N*−*p*_| = **|K**_*p*−*N*_|), the contribution of each fundamental sublattice (defined by a unique **|K**_*p*_|) can be expressed as:

(4) $$\begin{array}{ll} C_{p}\left(m,n\right) & =\sin\left(Tp\right)\left[\underset{\left(m,n|m-n=p\right)}{\sum }\sin\left(LTp+K_{p}\cdot r\right)\right.\\ & \;+\left.\underset{\left(m,n|m-n=N-p\right)}{\sum }\sin\left(LTp-K_{N-p}\cdot r\right)\right] \end{array}$$

where $\sum_{\left(m,n|m-n=p\right)}\sin\left(LTp+K_{p}\cdot r\right)$ runs over all pairs of interfering waves (*m*, *n*) that contributes to the same sublattice order *p*. For instance, the *p* = 1 sublattice is formed by the interference of all adjacent waves, while the *p* = 2 sublattice is formed by the interference of all next-nearest-neighbor waves. Equation (4) explicitly reveals that the total spin texture is the accumulation of every sublattice *C*_*p*_ over all possible values of *p*. The existence of each sublattice is governed by the factor $\sin\left(Tp\right)$ , which allows us to predict and explain the annihilation of certain sublattices.

### 2.2 Annihilation condition and number of sublattice

The sublattice structure for quasicrystal is not a fixed quantity and is influenced by multiple factors, which can be systematically suppressed or annihilated due to topological constraints. Two primary annihilation mechanisms are identified as:

(1) Geometric Annihilation: This is a trivial suppression effect arising from the geometry of the wavevector space, which occurs when the wavevector has zero amplitude. From Equation (3), this condition meets when $\sin\frac{\pi p}{N}=0$ , which implies that *p* is a multiple of *N* (including *p* = 0). These terms correspond to the central point in Fourier spectrum and do not contribute to the spin texture. As this effect is solely determined by geometric factors and is independent of the light’s topology, we term it geometric annihilation.
(2) Topological Annihilation: The second mechanism is a more profound, non-trivial effect arising from destructive interference governed by the topological properties of the electromagnetic fields. Similar to destructive interference, certain sublattices may vanish in the superposition process. The factor sin(*Tp*) dominates the existence of each sublattice. And the phase term *LTp* + **K** ⋅**r** may vanish within a pair of waves propagating in opposite directions, effectively erasing it from the total spin texture. It can be solved from the sublattice Equation (4), which leads to a selection rule where only sublattices with specified indices *p* are annihilated. For a pair of counter-propagating waves, the destructive interference yields:

(5) $$\sin\left(LTp+K_{+}\cdot r\right)+\sin\left(LTp-K_{-}\cdot r\right)=0$$

where **K**_+_ and **K**_-_ represent two oppositely propagating wave vectors. The destructive interference condition is solved as: *p* = *k*⋅*g* (*k* is an integer), which is governed by a key parameter, *g = N*/2*L*. This means a sublattice is annihilated if *p* is a multiple of the parameter *g*. Since this annihilation is topological in nature, which is closely related to topological charge value *L*, we term this process topological annihilation.

Generally, the number of sublattices is dependent on the number of possible values of *p* within the boundary $p\in \left[1-N,N-1\right]$ , while the degeneracy of wavevectors **|K**_*p*_| = **|K**_−*p*_| = **|K**_*N*−*p*_| = **|K**_*p*−*N*_| would exclude symmetrical values. For odd *N*, the parameter *g* cannot take integer value, and each wave with different vector amplitude contributes to global texture. The number of vector sets is:

(6) $$M_{\text{odd}}=\frac{N-1}{2}$$

For even *N*, the number of allowed sublattices is reduced by the topological annihilation. By excluding the annihilation solutions, the number of existing vector sets *M*_even_, can be obtained by subtracting the number of annihilated modes *M*_ann_ from the total possible modes:

(7) $$M_{\text{even}}=\frac{N}{2}-1-M_{\text{ann}}\left(N,L\right)$$

**Figure 3:**
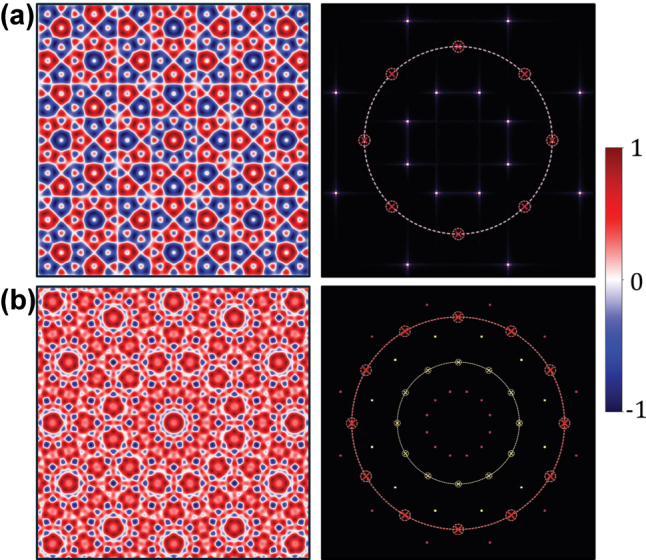
Verification of the topological annihilation mechanism. Spin textures (left panels) and their corresponding Fourier spectra (right panels) for (a) *N* = 8, *L* = 2 system and (b) *N* = 12, *L* = 3 system. The Fourier spectra show an absence of the corresponding wavevector rings, which is indicated by the dotted circles marks.

Equations (6) and (7) enable a priori determination of a spin quasicrystal’s structural complexity directly from the number of interfering waves *N* and the topological charge *L* of the incident beam. We verify the annihilation in spin quasicrystals by examining two non-trivial cases in Figure 3. For the *N* = 8, *L* = 2 system (*g* = 2), the unique sublattice orders are identified as *p* = 1, 2, 3. Within the range, the only multiple of *g* is *p* = 2. As shown in Figure 3(a), the wavevector rings corresponding to the predicted annihilated modes are absent, which are highlighted by the dotted circle marks. For the more complex *N* = 12, *L* = 3 system (*g* = 2), the annihilation occurs for both *p* = 2 and 4 within their range of {1, 2, 3, 4, 5}, as shown in Figure 3(b).

## 3 Skyrmionic textures in spin quasicrystals

The sublattice configuration in spin quasicrystals enables topological transformation, allowing the complex quasicrystalline structure to be reconfigured into a lower-order, periodic lattice that retains skyrmionic textures such as a skyrmion or meron lattice. This transformation is achieved through phase modulation, effectively filtering out specific points in frequency domain.

### 3.1 Topological transformation of spin quasicrystals

Photonic spin lattices are in general multiple superposition of adjacent wave interferences, where individual interference patterns could be split out. Triangle meron lattices, square meron lattices and hexagonal skyrmion lattices are formed under special symmetry constraint. For quasicrystals under other symmetry, it can be considered as hybrid composition of periodic lattices.

**Figure 4:**
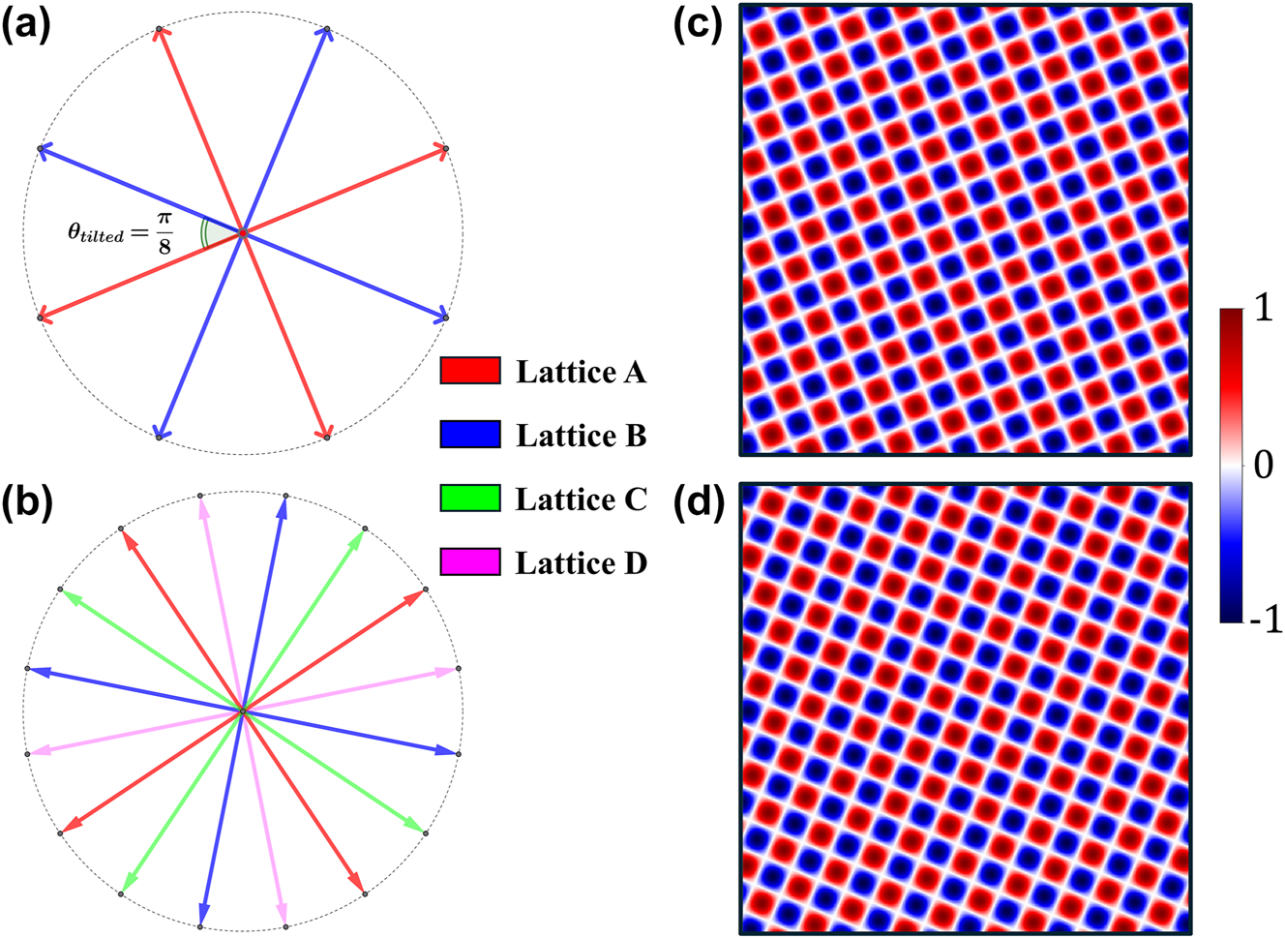
Decomposition of spin quasicrystals into periodic lattices. (a-b) Wavevector diagrams for the *p* = 1 sublattice of (a) an *N* = 8 system and (b) an *N* = 16 system. The vectors can be grouped into orthogonal sets. For *N* = 8, there are two sets of four orthogonal vectors. For *N* = 16, there are four sets. (c-d) The spin textures generated by activating only (c) Lattice A or (d) Lattice B. Each is a pure square meron lattice, mutually rotated by *θ*_tilted_ = *π*/8.

We take spin quasicrystal with octagonal symmetry (*N* = 8, *L* = 1) as an example. From the decomposition analysis in Sec. 2.2, it is composed of multiple sublattices without annihilation (*p* = 1, 2, 3). To demonstrate the principle of topological transformation, we focus on the first-order sublattice (*p* = 1). As illustrated in the wavevector diagram in Figure 4(a), its eight constituent wavevectors pointing along different directions can be grouped into two distinct sets of four orthogonal vectors, denoted by lattice A (red) and lattice B (blue). These two sets are mutually rotated by an angle of *θ*_tilted_ = *π*/8. The spin textures generated by individual lattice A and B are depicted in Figure 4(c) and (d), demonstrating two periodic 4-fold meron lattices distinguished by a relative angular twist *θ*_tilted_. This reveals that the sublattice under octagonal symmetry is essentially a superposition of two meron lattices with square symmetry. This inherent nature is not limited to *N* = 8 system. Higher-order even-*N* systems exhibit similar reducibility. For instance, the *p* = 1 sublattice of *N* = 16 quasicrystal can be decomposed into four meron lattices. The synthesis wave vector distribution is shown in Figure 4(b), which contains 16 equally separated wavevectors, together with a titled angle of *θ*_tilted_ = *π*/16. This inherent reducibility is the crucial feature that enables their selective manipulation.

The decomposition and transformation of quasicrystal enable selective activation or suppression of individual constituent sublattices through controlled destructive interference. This can be implemented by applying a targeted phase modulation before the excitation of SPP. Experimentally, this pre-excitation modulation can be realized by a specially designed polygonal excitation slit with shifted edges to generate appropriate phase differences between surface waves. Specifically, we apply an additional, alternating phase ± *α* to the surface waves propagating along each direction. The phase modulation profile alters the interference conditions for each lattice independently. One lattice is suppressed when its interference terms average to zero, yielding a similar relation to Equation (5):

(8) $$\sin\left(LTp+K_{+}\cdot r+\alpha_{+}\right)+\sin\left(LTp-K_{-}\cdot r+\alpha_{-}\right)=0$$

where *α*_+_ and *α*_−_ are the applied phase on each wave. Due to the alternating phase setup, opposite wave shares the same applied phase. This leads to an annihilation condition for the phase: *α*_+_ = *α*_−_ = −*LTp* + *kπ*.

For *N* = 8 and *L* = 1 system, the annihilation phase is calculated as $\alpha_{+}=-\frac{\pi }{4}+k\pi$ (*k* is an integer). The four orthogonal wave vectors in one group disappear with the import of phase *α*_+_. At the same time, the topology of another group of vectors with phase *α*_−_ remains unchanged, forming the 4-fold symmetry meron lattice. By splitting out the two vector groups and eliminating one group, an 8-fold quasicrystal is transformed into 4-fold square meron lattice in the sublattice domain. The resulting sublattice is periodic, featuring a significantly simplified and ordered topological structure.

### 3.2 Engineering complex lattices and formation of meron bag

The principle of selective sublattice annihilation provides a general tool for engineering complex spin textures. While the spin quasicrystal in *N* = 8 system behaves like a simple on/off photonic switch, the richer structure of high-order systems unfolds the wider potential of phase modulation. In a higher order *N* = 16 system, the *p* = 1 sublattice is composed of four distinct orthogonal *N* = 4 lattices each rotated by an angle of *θ*_tilted_ = *π*/16 relative to its neighbor, providing an abundant parameter space for manipulation. Instead of functioning as a simple switch, the phase modulation can act as a multi-channel selector in high-order quasicrystals.

**Figure 5:**
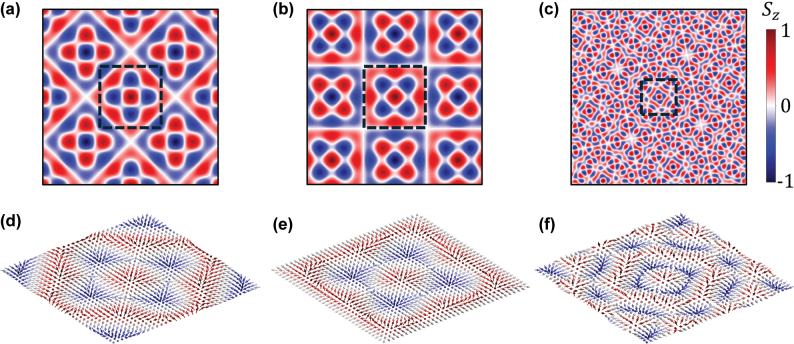
Engineering complex lattices for the generation of meron bag. (a-c) The synthesis longitudinal spin textures by activating (a) lattice A and B in Figure 4, (b) lattice C and D in Figure 4 and (c) lattice A, B and D in Figure 4. (d-f) The corresponding vector distribution for the areas highlighted in the (a-c). Each panel demonstrates the meron bag where a controlled number of topological units are packed into a single unit cell.

By designing a more sophisticated phase profile, we can selectively excite specific groups of vectors and construct their interference patterns, as depicted in Figure 5. For instance, by activating two sets of vector groups (e.g., lattice A and B), a hybrid meron lattice is generated (Figure 5(a)). The spin vector distribution (Figure 5(d)) reveals that the unit cell contains five meron cores with opposite topological charges, whose swirling textures are spatially displaced and entangled. This arrangement creates a quadrupolar-like distribution of in-plane spin angular momentum. Activating lattice C and D would generate similar meron lattice with a *π*/4 rotation (Figure 5(b) and (e)). The complexity can be further increased by activating three vector groups (lattice A, B and D), resulting in the even more complicated, yet ordered spin texture (Figure 5(c)). The unit cell consists of a densely warped array of meron cores, exhibiting a higher topological charge density, and a lower-order rotational symmetry compared to the former cases. The corresponding vector field exhibits multiple alternating vortices and anti-vortices of spin flow, a hallmark of high-density topological packing (Figure 5(f)). When all four sublattices are active, the full sublattice texture is recovered. Different compositions of vector groups result in distinct spatial arrangements and SAM distributions. This ability to pack a controllable number of topological units in a local unit cell gives rise to the formation of meron bag concept. A meron bag can be understood as a synthesized topological superstructure whose unit cell contains a chosen number of meron cores, which is different from the conventional meron lattice. The meron lattice exhibits a simple, periodic alternation of spin vectors across the entire structure. In a meron bag, a more complex multi-level alternation of the spin vectors occurs within the confined space of a single supercell. This strategy transcends traditional lattice formation, not by manipulating single skyrmion, but by controlling the number and arrangement of multiple skyrmions in a local unit cell, allowing for on-demand control of local topology and multi-dimensional topological data storage.

### 3.3 Symmetry principles for topological programming

The reconfigurability of sublattice of a spin quasicrystal is not a universal property but is strictly governed by the symmetry of system. While systems like *N* = 8 and *N* = 16 exhibit substantial applicable properties, others, such as those with odd-*N* symmetry, are stable and trivial, showing rare relations with periodic lattice. Here, we establish the fundamental symmetry principles that determine the reconfigurability of quasicrystals.

The analysis reveals the potential for an *N*-fold quasicrystal sublattice to be programmable, which means that it can be crafted into a multichannel selector and transformed into crystallographic lattice, and is constrained by three fundamental principles:

(1) Geometric Decomposability: The symmetry of system *N* must be a multiple of the order *k* of its constituent elementary units (i.e., *N* = *M⋅k*). This principle ensures that the sublattice can be geometrically partitioned into a set of identical, lower-symmetry vector groups.
(2) Physical Controllability: The order *k* of elementary unit must be even. This is a deterministic requirement, as the phase-modulation technique fundamentally relies on manipulating the interference of “opposite propagating wavevector pairs”, a symmetric structure that only even-*k* systems possess. Odd-*k* systems, lacking this symmetry for annihilation, act as irreducible interference units.
(3) Crystallographic Periodicity: To synthesize a periodic lattice, the order *k* of elementary unit must obey the crystallographic restriction theorem, limiting it to set {3, 4, 6}. This makes sure that the synthesized product belongs to non-trivial, tessellating crystal lattice.

This principle naturally describes all quasicrystals into two functionally distinct groups. The first group, monolithic quasicrystals, comprises all systems that violate this principle, including all odd-*N* quasicrystals and certain even-*N* systems (e.g., *N* = 10, 14…). Their symmetry is irreducible, meaning their sublattices cannot be decomposed into controllable even-*k* crystallographic units. This irreducibility is rooted in the structure of their wavevector space, which lacks the pairwise inversion symmetry condition essential for the control scheme. As a result, they form highly stable, self-contained topological structures. They can be considered as elemental entities in quasicrystal system, which is difficult to implement switched off effect or converting sublattices into simpler lattices using the phase modulation scheme. The second group, programmable quasicrystals (*N* = 8, 12, 16…), are those that follow this principle. Their reconfigurability stems from the inherent symmetry degeneracy. For instance, in sublattice domain, an *N* = 8 system can be considered as two superimposed 4-fold merons. This degeneracy in the wavevector space provides distinct channels that can be individually addressed. The phase engineering acts as a selective perturbation that breaks this degeneracy, forcing the system to collapse into a chosen, lower-symmetry configuration. It is the broken symmetry of programmable quasicrystals that unlocks their potential for topological programming.

## 4 Conclusions

In conclusion, we have demonstrated the generation, decomposition and synthesis of spin quasicrystals. By establishing a universal formula for the generation of spin quasicrystal, we predict the structural complexity. Based on the theoretical model, we realized the topological transformation at the sublattice level via phase modulation. We have shown that this principle can be generalized to more complicated systems, enabling the synthesis of novel complex lattices which do not exist in nature. By selectively activating the lattice group, meron bag configurations – a concept for engineering dense topological charge densities by packing a controllable number of topological units into a single, reconfigurable unit cell, are generated. The results provide not only a novel insight for the design and analysis of complex quasicrystal systems but also open a new avenue for the development of reconfigurable topological photonic devices. The ability to dynamically control the topological textures could be pivotal for future applications in high-density optical information storage, programmable meta surfaces, and parallelized nanoparticle manipulation. Looking forward, these findings pave the way for enormous areas. Exploring the application of this concept to other physical systems, such as exciton–polaritons or acoustic waves, could unlock a new family of programmable topological materials.
